# Long term functional outcome evaluation in post flexor digitorum profundus tendon zone I rupture repaired by palmaris longus tendon grafting augmented with human amniotic membranes and adipose derived mesenchymal stem cell: A case report

**DOI:** 10.1016/j.ijscr.2023.107960

**Published:** 2023-03-05

**Authors:** Heri Suroto, Benedictus Anindita Satmoko, Twindy Rarasati, Tabita Prajasari

**Affiliations:** a Orthopedic and Traumatology Department, Faculty of Medicine, Airlangga University/Dr. Soetomo Academic General Hospital, Surabaya, Indonesia; b Faculty of Medicine, Gadjah Mada University, Yogyakarta, Indonesia

**Keywords:** Flexor digitorum profundus tendon rupture, Regenerative therapy, Tendon healing, Human amniotic membrane, Adipose mesenchymal stem cell, Case report

## Abstract

**Introduction and importance:**

The sport climbing has many complex maneuvers of the hand producing many potential injuries in flexor digitorum profundus tendon (FDPT). The late management due to an athlete high demand on competition makes the complication of retracted tendon and adhesion tend to occur. We provide the long terms functional outcome in FDPT zone I rupture repaired by palmaris longus (PL) tendon grafting augmented with human amniotic (hAM) and adipose derived mesenchymal stem cell (ASCs).

**Case presentation:**

We present a case of a 31-years old male sport climbing athlete with excruciating pain in the right middle finger due to an injury at distal phalangeal area occurred two months earlier. Intraoperatively, Bruner's incision was performed for exploration. A modified Kessler suture technique with running sutures around the sutured stump was used. We slightly overcorrected tension between PL and FDPT distal stumps. We shielded the distal and proximal sutured areas with hAM augmented with ASCs. The result was remarkable as he could return to competitive sport.

**Clinical discussion:**

Zones I and II have a high adhesion risk due to their complex structures. In the case of the PL tendon graft, the sutured stump lies in these zones which can affect outcomes. An HAM augmented with ASCs has an anti-adhesive property that allows smooth gliding of the tendon FDPT on two sutured stump junctions, as well as stimulating the tendon to produce tenocytes, which accelerates tendon healing.

**Conclusion:**

The combination of our technique and regenerative therapy effectively prevents adhesions and modulates tendon healing.

## Highlights

• The untreated flexor tendon rupture causes tendon and muscle degeneration.
• PL tendon graft provides sutured stump lying on high-risk adhesion zones I and II.
• hAM augmented with ASCs can regenerate tenocytes and act as an antiadhesive.
• The regenerative therapy showed remarkable results in this report.

## 1 Introduction and importance

Flexor tendon injuries account for 40 % of all climbing-related injuries, especially closed ruptures that can significantly reduce a climber's specific abilities. Due to their frequent use in this sport, the ring and middle fingers are the most commonly affected [1]. An injury to the closed zone I of the flexor digitorum profundus tendon (FDPT) is frequently misdiagnosed, resulting in a delay in treatment. Without proper repair of the FDPT, the distal interphalangeal joint (DIPJ) loses its bendability, resulting in hyperextension, impaired pinching, and poor gripping [2].

Surgical repair of a ruptured flexor tendon is necessary since approximation facilitates the healing process [3]. Around 30 % of cases of adhesion formation are associated with significant disability. The objective of repair surgery is to restore finger function and prevent adhesions and scarring [4].

An ideal barrier to prevent these complications has been considered to be the human amniotic membrane (hAM) which promotes the wound healing process through collagen stimulation leading to reducing peritendon adhesion after tendon repair surgery [5]. Furthermore, the use of adipose-derived stem cells (ASCs) promotes tissue regeneration by repairing tissue function and reducing tissue damage [6]. This paper aims to report the long-term outcome of FDPT zone I rupture repaired by palmaris longus (PL) tendon grafting augmented with hAM and ASCs in a sport climbing athlete. This case report has been reported in line with the Surgical Case Report (SCARE) 2020 Criteria [7].

## 2 Case presentation

**Fig. 1:**
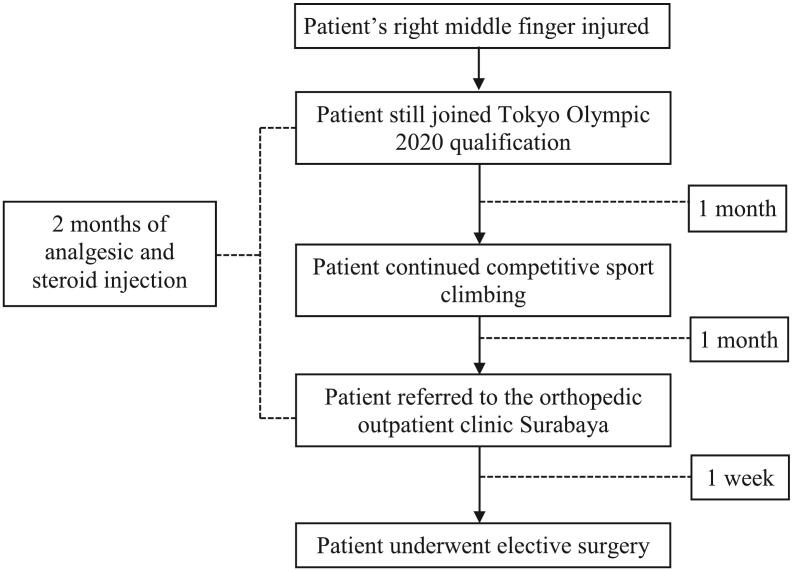
The timeline to surgery.

Mr. A, a 31-year-old male sport climbing athlete was referred to the orthopedic outpatient clinic with excruciating pain in the right middle finger due to an injury in the distal phalangeal area. He was unable to flex the DIPJ of the right middle finger, hence he could not continue the sport climbing competition. Prior to the trauma, the patient was an exceptional athlete who won many medals in the speed category of climbing. He sustained this injury two months earlier while climbing up a natural cliff. He used his right middle finger to hold on to the point pocket while climbing the 4 m cliff. In response to a sudden loss of footing, his right middle finger became trapped in the point pocket, creating an audible pop. He was treated with an ice pack due to the swelling, pain, and inability to flex caused by the injury. Following several analgesic and steroid injections at a Yogyakarta orthopedic outpatient clinic for one month, the patient was referred to a Surabaya orthopedic outpatient clinic. Fig. 1 illustrates the timeline from accident to surgery.

He had no previous trauma to the finger and no history of diabetes mellitus or other chronic disease. The physical examination showed positive result of FDPT test and negative result of sublimus test. The finger flexion cascade revealed that the right middle finger resting position was out of alignment. We did not find any deformities or wound scars on his right middle finger. During palpation, there was no palpable bulging along the FDPT with intensifying pain felt from the distal interphalangeal joint (PIPJ) to the metacarpophalangeal joint (MCPJ). The physical examination results can be seen in Table 1. It was found that the right middle finger was neurologically and vascularly normal. Ultrasonography (USG) revealed a rupture about 1 cm from its insertion in the right middle finger, and a retracted proximal stump in zone II. There were no fractures in the distal, medial, or proximal phalanges on plain radiographs. USG, radiographs, and clinical pictures were unavailable. The patient was diagnosed with FDPT rupture in zone I of the 3rd digit of the right hand.

**Table 1:** A three-year functional assessment of the hand.

Evaluation	VAS	Active ROM		TAM	% TAM	DASH score
		Flexion	Extension			
Before surgery	8			117°	41 % (poor)	61.7
DIPJ		0°	0°			
PIPJ		27°	0°			
MCPJ		90°	10°			
2 weeks after surgery	4			193°	68 % (fair)	35
DIPJ		28°	0°			
PIPJ		77°	15°			
MCPJ		90°	0°			
1 year after surgery	2			225°	91 % (excellent)	0.8
DIPJ		45°	0°			
PIPJ		90°	0°			
MCPJ		90°	0°			
3 years after surgery	0			234°	95 % (excellent)	0
DIPJ		54°	0°			
PIPJ		90°	0°			
MCPJ		90°	0°			

Note: DIPJ: distal interphalangeal joint; PIPJ: proximal interphalangeal joint; MCPJ: metacarpophalangeal joint; VAS: visual analogue score; ROM: range of motion; TAM: total active motion; %TAM: percentage of TAM; DASH: Disability of Arm, Shoulder and Hand (DASH).

### 2.1 Human amnion membrane and adipose derived MSC characteristic

**Fig. 2:**
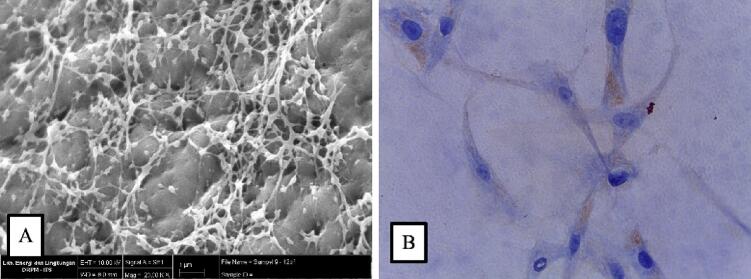
The picture of (A) the ASCs distributed evenly on hAM as scaffold by SEM and (B) the CD105 expression seen as brown-colored cytoplasm in >50 % cells. (For interpretation of the references to colour in this figure legend, the reader is referred to the web version of this article.)

We used hAM from Surabaya Cell and Tissue Bank for this procedure. ASCs derived from the other human lipid source were prepared by the Surabaya cell and tissue bank using standard allogenic mesenchymal stem cell preparation. As a result of scanning electron microscope spectroscopy (SEM), we obtained a picture of ASCs attached to hAM evenly distributed (Fig. 2A). In our procedure, the hAM which acted as a scaffold had an average pore diameter of 1.9 ± 0.3 μm. Furthermore, the ASCs had a concentration of 1.533 × 106 cells/mL. Cytotoxicity evaluations showed that the hAM seeded by ASCs had no toxicity to living cells, which its living cells increased five times higher than before ASC seeding. The CD105 expressions were shown in Fig. 2B.

### 2.2 Surgical procedure

We performed elective surgery under peripheral nerve block anesthesia. The patient was positioned supine on a hand table with clear exposure of the right lower arm and manus. We performed Bruner's proximal incision to explore the FDPT proximal stump and continued to zone I. The FDPT proximal stump was found to have receded to zone II. Its distal stump was located about 1 cm from the insertion site. It was noted that the annular and cruciate ligaments did not rupture. In order to fill the defect, we harvested 10 cm of PL tendon from the wrist area. We folded the graft in half to equalize the thickness of the FDPT proximal stump. A modified Kessler sutures were used for the FDPT and PL proximal stumps, enhanced by running sutures. A tension strength test was performed on the suture to determine its strength.

**Fig. 3:**
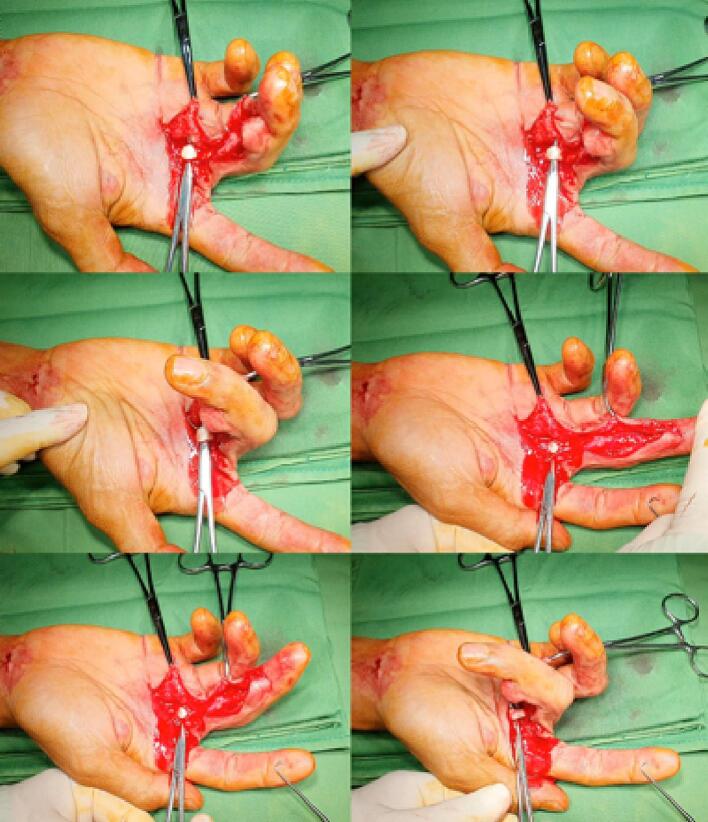
The intraoperative documentation showed the ROM, strength, and stability test of the repaired FDPT producing a full and stable ROM and adequate strength in the PIPJ and DIPJ.

**Fig. 4:**
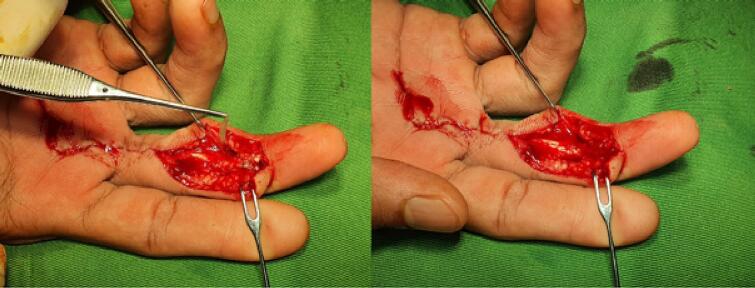
The hAM augmented with ASCs was applied to shield the distal and proximal sutured stump.

We inserted this tethered tendon into the A2 annular ligament via the camper chiasm and connected it to the distal stump of FDPT seen in the proximal A5 annular ligament. Based on the finger flexion cascade, we slightly overcorrected tension between PL and FDPT distal stumps. A modified Kessler suture technique with running sutures around the sutured stump was used. A monofilament non-absorbable 4.0 suture was used for the core suture, while a monofilament non-absorbable 6.0 suture was used for the running suture. By pulling the repaired FDPT in zone II, we tested it for ROM, strength, and stability (Fig. 3). As shown in Fig. 4, we shielded the distal and proximal sutured areas with hAM augmented with ASCs. There was no splint on the finger after closing the incision and dressing it with freeze-dried amniotic membrane. The first author performed this procedure in 1 h and 30 min.

(Source: internal documentation)

(Source: internal documentation)

### 2.3 Post-operative

After surgery, passive and active rehabilitation was provided without the use of a splint to allow all fingers to be flexed and extended early as long as the pain was manageable. A two-week postoperative evaluation revealed improvement in middle finger function. Some limited ROM was observed during the evaluation, but the finger flexion cascade was aligned to acceptable levels.

The rehabilitation program continued with active-progressive ROM exercise for 6 weeks. Due to depression, the patient was unable to comply with the rehabilitation program. The impact of this injury on his career was substantial because sport climbing relies on the strength and stability of the fingers. After three months of absence from the clinic, the patient returned to begin rehabilitation. Active ROM exercises with resistance, such as grasping the rehabilitation ball and exercising finger extensors with rubber bands, were provided for the patient. After one-year, middle finger movement and function were near normal with more power than previous evaluation.

**Fig. 5:**
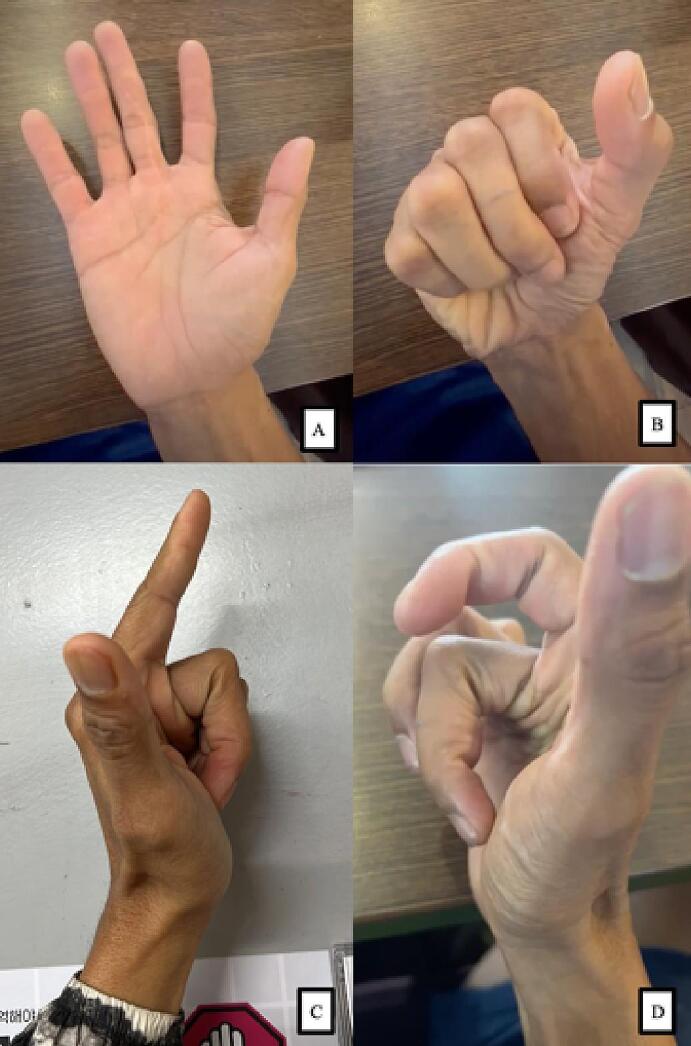
The three years post-operative evaluation elaborated the affected hand in (A) the full extension and (B) the full flexion of the fingers, in addition the documentation of normal left finger (C) compared to the affected fingers (D) in full flexion.

The patient returned to sport climbing training and competition in the male speed category. As a result of his consistency and perseverance, he won the male speed category at the 2022 International Federation of Sport Climbing World Championships. A clinical evaluation revealed that his finger function was within the normal finger cascade without any restrictions at his last follow up 3 years after surgery (Fig. 5). The post-operative evaluation of hand function is provided in Table 1.

(Source: internal documentation)

## 3 Clinical discussion

A higher adhesion risk might be occurred more likely in zone I or II primary and secondary surgery because of its complicated structure within these zone [8]. Even though there are many strategies applied to avoid adhesion formation in the repair site of tendon rupture, the poor results in zone I and II with the more chances of adhesion formation were reported by other several authors [9], [10]. Several methods have been developed for preventing peritendon adhesions and improving tendon healing quality following surgical repair, including improved suturing techniques and reconstruction of tendon sheaths [11]. Many studies report unsatisfactory results with different substances for inhibiting inflammation or synthetic or biological materials for shielding the sutured tendon [12], [13].

The hAM has low immunogenicity as it contains human HLA-G which is an immunosuppressive factor and the Fas ligand. This biological barrier also reduces protease activity by secreting tissue inhibitors of metalloproteinases and repressing transforming growth factor beta, the activating factor for fibroblasts, resulting in an anti-adhesive and anti-scarring effect [14]. The hAM promotes the wound healing process through collagen stimulation leading to reducing peritendon adhesion after tendon repair surgery [15]. Prakash et al. confirmed that hAM wrapping provides smooth tendon gliding demonstrated with USG. The hAM wrap at the repair site creates a tunnel that emanates from the hAM transformation to form a similar structure to a tendon sheath [5]. Furthermore, several studies have demonstrated that ASCs promote the organization of collagen fibers in tendon injury [16], [17]. They have been found to differentiate into tenocytes, thereby providing substantially more capability in the clinical application of tendon regeneration and repair. Through the secretion of cytokines and growth factors, ASCs facilitate tissue regeneration and minimize tissue damage. ASCs enhance biomechanical properties and the arrangement of collagen fibers. The additional advantage of ASCs reported by Kokubu et al. is neovascularization induction in the early phase of tendon healing [18]. Shen et al. studies showed that ASCs promoted a regenerative and anti-inflammatory M2 macrophage phenotype and affected the tendon extracellular matrix remodeling, angiogenesis and cell survival [19].

In this case, the FDPT stump degenerated and retracted to zone II of the flexor hand, whereas the distal stump remained in zone I. The problem was resolved by inserting tendon grafts into a five-centimetre gap. However, adhesions could form at suture stump junctions in zones I and II. In addition, the late diagnosis of this FDPT rupture complicated the surgical repair and worsened the prognosis. The PL tendon was harvested twice longer than the tendon gap and folded into half to achieve a similar size and strength to the FDPT. A slight overcorrection of FDPT tendon tension compensated for muscle atrophy after prolonged tendon rupture. It was crucial to restore the middle finger to its previous condition after an injury to return to competitive sport climbing. The use of hAM augmented with ASCs combined with early mobilization of the injured hand without the use of a splint can prevent adhesion at the repaired site. As result, He was able to resume competitive sport climbing due to nearly normal 3rd digit function and gliding. This report has some limitations such as not providing the picture of USG examination because the patient lost the print results and the absence of the clinical picture before surgery that could show the condition of the finger before incision.

## 4 Conclusion

The FDPT rupture repair surgery in a sport climbing athlete requires specific techniques to achieve a satisfactory outcome. The hAM augmented with ASCs may be used as a biological barrier to prevent adhesion formation, which is capable of promoting differentiation of tenocytes and modulating tendon healing, particularly in the zone I and zone II flexor tendon. The results of this report support prior studies demonstrating the effectiveness of hAM augmented with ASCs as a regenerative therapy for tendon healing.

## Patient consent

Written informed consent was obtained from the patient for publication of this case report and accompanying images. A copy of the written consent is available for review by the Editor-in-Chief of this journal on request.

## Patient perspective

I was losing hope to continue competitive sport climbing. However, the physician's surgical technique and rehabilitation support me until I won a gold medal in sport climbing championship.

## Ethical approval

Ethical approval was waived at our institution.

## Funding

This case report received no specific grant from any funding agency in the public, or non-profit sector.

## Author contribution

Heri Suroto involved in conceptualization, case presentation, data collection, elaborating the surgical technique, main guidance for write up, reviewing, and editing the manuscript.

Benedictus Anindita Satmoko involved in conceptualization, case presentation, data collection, writing, reviewing, and editing the manuscript.

Twindy Rarasati involved in data collection and writing the manuscript.

Tabita Prajasari involved in data collection, conceptualization, reviewing, and editing.

## Guarantor

Heri Suroto.

## Research registration number

Not applicable.

## Additional information

This case report has been presented at Indonesia Association for Upper Limb & Microsurgery (PERAMOI) regional meeting 2022.

## Declaration of competing interest

The authors have no conflicts of interest to disclose.
